# Training Self-Administered Acupressure Exercise among Postmenopausal Women with Osteoarthritic Knee Pain: A Feasibility Study and Lessons Learned

**DOI:** 10.1155/2012/570431

**Published:** 2012-10-23

**Authors:** Yan Zhang, Chwan-Li Shen, Kim Peck, Jean-Michel Brismée, Susan Doctolero, Di-Fan Lo, Yik Lim, Lixing Lao

**Affiliations:** ^1^Department of Family and Community Medicine, Texas Tech University Health Sciences Center, 3601 4th Street, STOP 8143, Lubbock, TX 79430, USA; ^2^Department of Pathology, Texas Tech University Health Sciences Center, Lubbock, TX 79430, USA; ^3^School of Allied Health Sciences, Texas Tech University Health Sciences Center Lubbock, TX 79430, USA; ^4^Clinical Research Institute, Texas Tech University Health Sciences Center Lubbock, TX 79430, USA; ^5^School of Nursing, Texas Tech University Health Sciences Center, Lubbock, TX 79430, USA; ^6^Department of Internal Medicine, Texas Tech University Health Sciences Center Lubbock, TX 79430, USA; ^7^Department of Family and Community Medicine, TCM Research Program, Center for Integrative Medicine, University of Maryland School of Medicine, East Hall, 520 W. Lombard Street, Baltimore, MD 21201, USA

## Abstract

*Background*. Osteoarthritis (OA) is more prevalent in women, particularly after menopausal age. Women are more likely to seek complementary and alternative medicine (CAM) approaches. We examined the feasibility of training self-administered acupressure exercise and assessed its impact on OA symptoms among women with knee OA. *Methods*. Thirty-six eligible postmenopausal women were randomly assigned in the acupressure exercise group (*n* = 15) or the control group (*n* = 21) for 12 weeks. Feasibility outcomes (e.g., compliance and adverse effects) and clinical outcomes (e.g., pain, stiffness, and physical function) were assessed. Data were collected at baseline, 6 weeks and 12 weeks. Both per-protocol and intention-to-treat analysis were employed. *Results*. The training materials were well received. The feedback from participants suggests that self-administered acupressure exercise is easy to learn and safe to perform at home, although no statistically significant results of the clinical outcome were observed. Our findings didn't reveal superiority or inferiority of acupressure compared with usual care. *Conclusion*. Acupressure exercise is feasible to be trained among postmenopausal women with knee osteoarthritis. Due to the limitations of this study such as small sample size and high attrition rate, acupressure's efficacy needs to be further explored in larger scale studies with more rigorous design.

## 1. Introduction

Osteoarthritis (OA) is a common progressive joint disease and major cause of disability in the aging population in the United States, especially among women [1, 2]. Incidence of OA were 1.7 times higher in women than in men after the age of 50 years, around the same time when women experience menopause transition [3]. Of all symptomatic OA, knee OA is the most prevalent, affecting about 12% (4.3 million) of American adults aged 60 years and older, and 16% of aged 45 years and older [4, 5]. Similarly, in the elderly, the new onset of knee OA is more common in women [3], who have nearly twice increased knee OA incident risk compared to men [2]. 

Currently, no curative therapies exist for OA. The foundation of conventional treatment includes analgesia, physiotherapy, and joint replacement if indicated [6, 7]. Those treatments have limited effectiveness, and toxicities related to pharmacologic medications often cause unwanted side effects such as pain, muscle weakness, lack of stamina, and loss of function or even dangerous side effects such as gastrointestinal toxicities and increased cardiovascular risk [8–12]. A review of OA conservative treatment modalitiessuggests that applying complementary therapies concurrently may be more effective [1]. Acupuncture, massage therapy, and certain other complementary and alternative medicine (CAM) interventions are utilized by OA sufferers and represent attractive, potentially effective options to manage pain and OA symptoms [13–21].

CAM is commonly used to manage joint and arthritis pain among persons with knee OA [22]. Previous reviews cited evidence-based effectiveness of acupuncture for OA in reducing pain [23, 24]. Acupuncture, as a CAM approach, has been widely used for treating pain including knee pain from knee OA [16–20, 25]. Another CAM approach, Swedish massage, was recently found effective in improving pain, stiffness, and physical functional disability of knee OA subjects [14, 15]. Acupressure shares some characteristics of both acupuncture and massage. Acupuncture and acupressure use the same acupoints (acupuncture points, sometimes called trigger or active points) for treatment purpose, but acupuncture employs needles, while acupressure uses the fingers to press acupoints on the surface of the skin to stimulate the body's natural self-curative abilities. Traditional Chinese medicine holds that certain channels called meridians in the human body regulate the flow of vital energy (called Qi) and it is the unbalanced flow of Qi that results in disease. Stimulation such as needling or pressing at the acupoints on the meridians is believed to open the channels and balance energy, thus restoring health to the body. In addition, mechanical pressure, such as massage and acupressure, has been known to decrease tissue adhesion, promote relaxation, increase regional blood circulation, increase parasympathetic nervous activity, increase intramuscular temperature, and decrease neuromuscular excitability [26]. 

Similar to acupuncture and massage, trained practitioners often administer acupressure, although the latter has the advantage that it can be used as a self-care approach at home. Self-administered acupressure, if proven feasible and effective, is convenient and inexpensive. A few researchers have investigated the usefulness of acupressure for knee pain [27], but to our knowledge, studies on self-administered acupressure protocol are scarce; therefore it is imperative to develop a standardized manual or protocol to guide participants to perform acupressure safely and accurately. 

We chose postmenopausal women with OA knee pain as our target population for three reasons. First, this group of women is reported having higher incidence and/or more severe symptoms of knee OA [2]. Secondly, for a clinical diagnosis of knee OA, participants have to report knee pain and possess three of six criteria; one of these being that they are older than 50 years [28]. Thirdly, women are of special interest because they are more likely than men to use CAM therapies [29, 30], and they frequently reported using CAM in conjunction with conventional medicine to manage chronic pain conditions [31]. Therefore, only postmenopausal women with knee osteoarthritis were included in this feasibility study. If acupressure demonstrates good training feasibility and positive results in improvement of OA knee symptoms among this group, we aim to promote it to other age groups of both genders.

The specific aims were twofold: (1) to examine the feasibility of training acupressure exercise among female patients with OA knee pain, and (2) to assess the impact of acupressure exercise on knee pain and other OA knee symptoms. We hypothesized that it would be feasible to teach study participants to self-administer acupressure exercise for treating knee OA and that self-administered acupressure exercise would decrease knee pain and improve physical function. 

## 2. Methods

### 2.1. Sample and Recruitment

This study was approved by Texas Tech University Health Sciences Center Institutional Review Board (IRB). Women with knee OA were recruited from clinic and community settings in a West Texas metropolitan area. Participant recruitment involved informational letters to patients with documented knee OA at the local family medicine and orthopedic clinic; IRB approved fliers and press release distributed at the clinic, local newspaper and TV station, regional organization newsletters, and institutional announcement website during a three-month period in the summer (May to July) of 2011. 

Volunteers who made contact with the project coordinator (SD) were screened for eligibility over the phone using a preparticipation survey which included self-reported height, weight, rating on a 10-point Likert pain scale, their current medications, and questions generated based on inclusion and exclusion criteria. Inclusion criteria were as follows: (1) women only, (2) age between 50 and 70 years, (3) body mass index (BMI) ≤35 kg/m^2^, (4) diagnosed as having OA of the knee for at least 6-month duration, (5) having knee pain, (6) having good to satisfactory general health rating, (7) had suffering from mild to moderate knee symptoms during most days throughout the past month; or, pain in the knee in the preceding 2 weeks ≥3/10 on a Likert pain scale from 1–10, (8) stable treatment with nonsteroidal anti-inflammatory drugs and analgesics in the previous month, (9) if receiving glucosamine, a stable dose for the past 2 months, and (10) willing and able to complete the study protocol. Exclusion criteria were as follows: (1) had intra-articular corticosteroid injection into the knees within 8 weeks preceding the study, (2) had knee injury and/or hand injury that prevent the participant to perform acupressure, (3) had knee or hip replacement, (4) prior or current treatment with acupuncture for knee pain, (5) autoimmune disease that caused joint pain such as rheumatoid arthritis and lupus, and (6) severe unstable chronic illness or terminal disease.

### 2.2. Design and Intervention

This study employed a randomized controlled nonblinded trial with one treatment arm and one control arm to examine the effect of 12-week acupressure exercise on pain, stiffness, and physical function in knee OA and quality of life among postmenopausal women. The 36 women were randomly assigned into either the acupressure or the control group by drawing the group membership in a sealed envelope.

**Acupressure Group**

**Figure 1:**
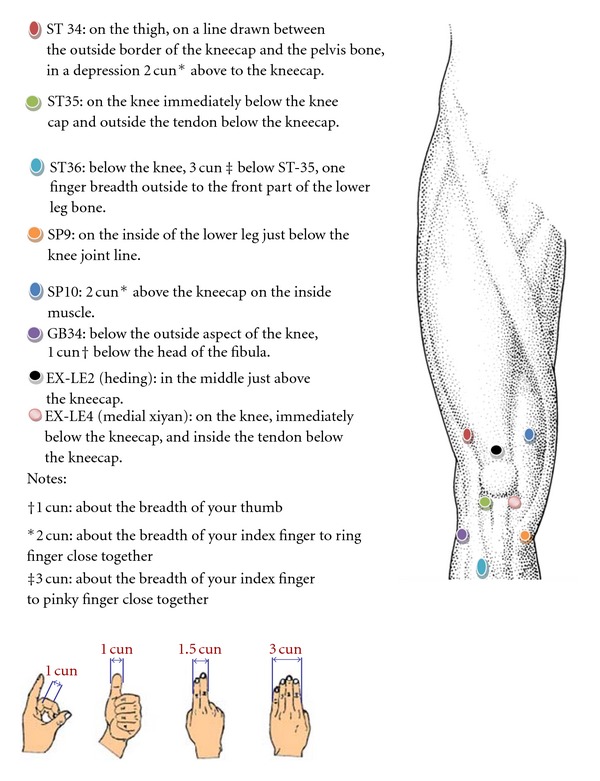
Acupoints used in the protocol.

Participants in the acupressure exercise group received two guided acupressure training sessions and one conclusion session during the 12-week study period. They were asked to continue their current usual care simultaneously. The training was conducted in a group format. The training material content was developed by a licensed acupuncturist (YZ) and all the training materials were reviewed, approved, and produced by the research team. A training kit including a demonstration DVD and handouts, along with an acupressure tool, was provided to each study participant. The DVD video demonstrated how to conduct the acupressure exercise step by step. The handout included a picture-illustrating acupressure step-by-step protocol, WARM (*warm-up* about 3 minutes, *acupressure* about 10 minutes, *rubbing kneecap* about 3 minutes, and *moving* your knee about 3 minutes), and an acupoint chart providing information on identification of the eight knee acupoint locations (i.e. ST34, ST35, ST36, SP9, SP10, GB34, EX-LE2, and EX-LE4, Figure 1). The WARM protocol was designed based on meridian theory that is the key principle of acupressure. Besides the core component acupressure, it incorporated necessary warm-up and cool-down phases that are needed when applying acupressure exercise. In brief, *warm-up* requires the participant to use one hand to form a claw shape to squeeze then move along the front thigh (i.e. quadriceps where meridians liver, spleen, stomach, and gallbladder locate). *Acupressure* session requires the participant to use the acupressure tool to press down the acupoints briskly then release with moderate pressure, repetitively for about 1 minute per point. The acupressure tool used in this study is a manual device called *Palmassager* that has three smooth round knobs to help provide stress on the point. *Rubbing the knee* requires the participant to use one palm to cover the kneecap, assisting with fingers, to lift the kneecap gently, and gently move the palm in a small circle. *Move the knee* requires the participant to extend and rotate leg while sitting at the edge of a bed or a chair with her leg hanging with the hands holding the thigh on both sides to keep the thigh stationary. Although the acupressure exercise itself requires about 20 minutes, we asked the participants to allow themselves 30 minutes for the whole protocol including preparation time each day. Participants were asked to perform this protocol at home five days a week for 12 weeks. Participants with bilateral knee pain were asked to apply the acupressure exercise on the knee reported to be more painful at the initial interview. Participants with pain in only one knee were asked to apply acupressure exercise on the knee with pain.

The 1st session (i.e. the initial training) was conducted at the beginning of the 1st week of the intervention. During this session, the project staff (YZ and SD) provided a brief introduction about acupressure, played the demonstration DVD, then went over each step in the training materials, and helped the participants with supervised hands-on practice to master the techniques. The training session was adjourned when all the participants expressed understanding and showed mastery of the techniques. During the 2nd session (i.e. the refreshing training) conducted in the 7th week of the intervention (i.e. half-way through this intervention), the project staff revisited the theory of acupressure and addressed issues raised by the participants during their past 6-week practice. Feedback regarding the training materials and at-home performance of acupressure was collected. Some participants voluntarily demonstrated how they conducted acupressure exercise at home with the acupressure tool and corrections were made when necessary. The 3rd session (i.e. the conclusion session) was conducted at the end of the 12th week. During this session, the trainer and investigators congratulated the participants for their completion of the study and awarded them the completion certificates. Continuing acupressure exercise at home was discussed based on their responses.

**Control Group**

Participants in the control group continued their current usual care and received no intervention during the same 12-week study period. At the end of this study, they were invited to the conclusion session during which they were offered the same training as the initial training in the intervention group and given the acupressure kit and the acupressure tool.

### 2.3. Outcome Measures

A project log recorded information regarding recruitment, data collection status, training attendance, and follow-up status. The participants were asked to contact the project coordinator if their current medication changed, or they had a procedure such as injection or surgery during the study period. Participants used an acupressure log to record the frequency, duration, time of acupressure and self-rating pain level at home.

The 24-question Western Ontario and McMaster's University Osteoarthritis Index (WOMAC) was used to measure subscales of pain (5 items), stiffness (2 items), and physical function (17 items) in knee OA [23]. The self-administered WOMAC is available in 5-point Likert-type format with highest scores indicating more severe impairment [32]. A negative change in WOMAC scores from previous time point indicates improvement whereas a positive change indicates worsening of symptoms. The WOMAC global score was computed as the unweighted mean of all 24 items. WOMAC scores were the primary outcome. 

The SF-36 survey (version 2) was used to measure health status and quality of life (QoL) [33]. The 36-question SF-36 includes the following eight subscales: physical functioning (PF), role limitations due to physical health (RP), bodily pain (BP), general health (GH), vitality (VT), social function (SF), role of limitations due to emotional health (RE), and mental health (MH). Two summary measures, the Physical Component Summary (PCS) and the Mental Component Summary (MCS) are also assessed. These scales are scored 1–100, with a higher score representing better functioning on the two summary measures and eight subscales. A negative change in SF-36 scores from previous time point indicates worsening whereas a positive change indicates improvement of symptoms. SF-36 was the secondary outcome.

### 2.4. Data Collection

Upon IRB approval, all questionnaire data were collected at baseline, 6, and 12 weeks after the intervention started. The questionnaire data were collected in person at baseline and again at the end of 12 weeks for both acupressure and control group when they came to the sessions. A set of WOMAC and SF-36 questionnaires with prepaid returning envelopes were mailed to all the participants during the 6th week. 

### 2.5. Statistical Analysis

As the first study to explore self-administered acupressure's impact on OA knee symptoms, we used Cohen's effect size (ES) index to estimate the sample size [34]. A sample size of 50 participants with an expected attrition rate of 15% over 12 weeks of intervention was determined to produce a final sample size of 40 participants, and to provide 80% power to detect medium (ES index = 0.5) to large effective size (ES index = 0.8) at alpha level of 0.05. Deidentified data were analyzed by SPSS statistics (version 20, IBM Corp). Descriptive statistics (e.g. normality) for each relevant variable at baseline were determined to justify parametric or nonparametric methods. Continuous variables were presented in mean ± standard deviation (SD) format unless otherwise stated. Norm-based scoring of SF-36 was obtained from SF-36 software (version 2). Changes of the WOMAC and SF-36 scores between different time points were calculated. Repeated measure ANOVA model was used to assess the acupressure's impact over time among outcome variables meeting the ANOVA assumptions. Both per-protocol and intention-to-treat analyses were used to assess the noninferiority and superiority of the trial. Per-protocol analysis included only those patients who completed the treatment originally allocated. Due to the small size of the final analytic sample, nonparametric Man-Whitney *U* test was performed to assess the difference between the two groups among all clinical outcome variables. We used “last value carried forward” for missing observations in intention-to-treat analysis. The significance level was set at *P* < 0.05. Bonferroni adjustment was used for multiple comparisons; therefore significance levels were adjusted as *P* < 0.05/3 = 0.016 for WOMAC and *P* < 0.05/8 = 0.006 for SF-36, respectively.

## 3. Results

### 3.1. Feasibility

At the end of the recruitment stage, of the 221 who made contact with the coordinator for phone screening, 51 participants were eligible with 36 agreeing to participate. The most common reasons for ineligibility were, (1) BMI > 35 as follows: (2) recent history of steroid injection on the knees as follows: and (3) history of knee replacement. Each patient provided written informed consent prior to enrollment. 

Of all 36 participants, 21 were allocated to the control group and 15 to the acupressure participants. No statistical significant differences of participants' characteristics at baseline were found between the acupressure and control group (see Table 1).

**Figure 2:**
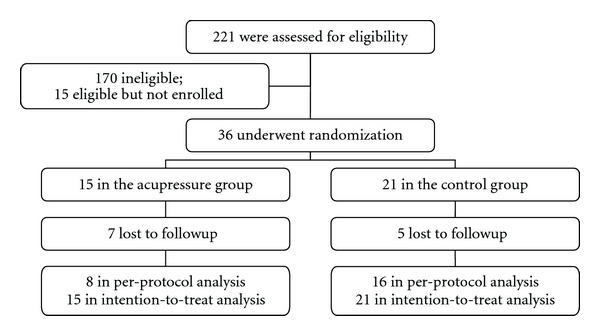
The flow of participant recruitment and retention.

By the end of study, 12 women dropped out from the study, 7 (47%) from the acupressure group, and 5 (24%) from the control group. Reasons of dropping out from the acupressure group included no time to do the daily logs and continue with the study (*n* = 3), a nonacupressure related fall (*n* = 1), a family emergency (*n* = 1), not finding acupressure improving her symptoms during the first couple of weeks (*n* = 1), and cortisone injection for the knee pain (*n* = 1). Reasons of dropping out from the control group included no longer being interested in the study (*n* = 3), or not showing up for the last data collection (*n* = 2).  Figure 2 shows the flow of recruitment and retention of the study. 

Seven acupressure group participants returned to the 2nd session, two verbally appraised acupressure as “helpful” in improving their symptoms, and one commented as indifferent (i.e. not getting better or worse). All expressed the 2nd session helped them to better master the techniques after exchanging information with peers and trainers. Verbal feedback during the 2nd session revealed that the training kit was well accepted with participants commenting that the handout was “easy to understand and follow.” The acupressure tool was preferred because it helped with accurate placement of pressure and avoided overuse of the fingers. No adverse event related to acupressure was reported. Very few participants completed the acupressure log; therefore the log content was not included in our analysis and results. 

### 3.2. WOMAC

No statistical differences were found between the two groups among all WOMAC subscale and total scores at baseline. Per-protocol analysis (Table 2) revealed that of the three WOMAC subscales, only physical function demonstrated normality at all three time points. Repeated measure ANOVA showed that the interaction between time and group membership had potential impact on physical function (sphericity assumed *F* = 4.44; *P* = 0.02) controlling for age, baseline BMI, and pain scores (Table 2). Mann-Whitney *U* tests indicated that physical function changes from baseline to 12 weeks were different between the acupressure and control group (*P* = 0.03), with the acupressure group showing greater improvement. However, the above results were not significant after Bonferroni adjustment (*P* > 0.016). No significant differences were found in the subscales of pain and stiffness. No statistically significant differences were found in intention-to-treat analysis (Table 3).

### 3.3. SF-36 (QoL)

No statistical differences were between the two groups all SF-36 subscale and total scores at baseline. Per-protocol analysis showed that subscale PF, RP, BP, and PCS at all three time points demonstrated normality. Repeated measure ANOVA revealed that the interaction between time and group membership had significant impact on PF (sphericity not assumed, Greenhouse-Geisser test used, *F* = 3.95; *P* = 0.04) controlling for age, baseline BMI, and pain scores. Mann-Whitney *U* tests showed that acupressure group had significant improvement on PF from baseline to 6th week (*P* = 0.02), and on VT from baseline to 6th week (*P* = 0.04) and from 6th to 12th week (*P* = 0.04), and on role of limitations due to EH (*P* = 0.02). However, the above results were not significant after Bonferroni adjustment (*P* > 0.006). No statistical significant changes of subscales RP, BP, and PCS over time were found. No statistically significant differences were found in intention-to-treat analysis. Due to the overall negativity and complexity of presenting eight subscales plus sum scores, no table is presented for SF-36.

## 4. Discussion

To our knowledge, this is the first investigation to assess the feasibility of training self-administered acupressure and to explore its effect on symptoms among women with knee OA. The feedback we received from the participants suggests that self-administered acupressure is easy to learn and safe to perform at home, although no statistically significant results of the clinical outcome were observed. Our findings did not reveal superiority or inferiority of acupressure compared with usual care. This suggests that self-administered acupressure exercise does not pose harm or risk to the participant's current condition. The negative clinical outcome findings can be accounted by multiple factors in the recruitment, data collection procedure, and analysis. Lessons learned from our study are worth sharing with peers interested in this field.

Qualitative review of feedback from the participants indicated that self-administered acupressure could be taught with the assistance of a training kit. Although it is still in its preliminary format, our training kit was considered very helpful based on the positive feedback from the participants. The majority of participants verbalized that the acupressure handout/video was “straightforward” and “easy to follow”. The potential importance of acupressure as a CAM approach treating OA knee pain is self-evident when compared to conventional pharmacotherapy with adverse side effects. Compared with acupuncture and massage, the acupressure management approach has the advantage of being administered by the patients themselves, which results in very low cost. Further comparisons between acupressure, acupuncture, massage, and exercise on the management of knee OA symptoms are warranted to assess the cost effectiveness. More research investigating a combination of low cost interventions that include controlled movements, such as Tai Chi [35], and acupressure, may be valuable as both interventions are self-administered and have demonstrated benefit to participants with minimum to moderate knee OA condition. On this note, we acknowledge that the acupressure exercise protocol we designed could be considered as a combination of acupressure and exercise. Future larger scale studies are needed to pinpoint the efficacy of each component of the acupressure exercise protocol. 

The first limitation involves the sample size and recruitment. Our study did not reach the expected sample size. A post hoc power analysis revealed that on the basis of the mean and standard deviation of WOMAC physical function baseline to 6-month score changes, between-groups comparison effect size observed in the present study (*d* = 0.38), the actual detectable power of our study was 0.29 given the sample size (*n* = 36) and observed variability. This was apparently underpowered. To increase the power in future studies, establishing the appropriate inclusive and exclusive criteria will be necessary. Our eligibility criteria may have been too strict to recruit sufficient participants from the target population. For instance, the criteria of BMI < 35 alone eliminated about half of the candidates. In addition, many participants contacted us to participate in the study as a last attempt to delay or avoid total knee arthroplasty indicating our study participants already have severe knee OA. These participants reported higher knee pain scores and may not have been most appropriate for this feasibility study. Compared to one of our previous studies of participants with knee OA pain [35], the participants in this study were less overweight (lower BMI scores) but had more severe symptoms (higher WOMAC scores). Our previous study showed that greater pain relief occurred in populations reporting mild or moderate symptoms of knee OA [35]. Our findings showed a trend of physical function improvement at the early stage (6 weeks) of acupressure intervention in spite of the worse condition of our participants when enrolled. This indicates that studies with mild or moderate knee condition participants may yield more significant results. Future studies including both genders, broader age range, mild or moderate knee OA, and no BMI restriction may be in a better shape detecting any potential outcome significance. 

The second limitation concerns the randomization method. A simple random drawing of group membership used in this study resulted in the uneven number of participants in each group. A computer-generated blocked random allocation method is recommended in future studies. For instance, participants can be block randomized using a permuted block design and stratified by gender, body mass index (BMI), and screened pain scores to ensure balance between the intervention and control groups. Although blinding was not conducted in this feasibility study, it is recommended that future studies add blinding to certain degree. For instance, if the whole acupressure exercise protocol demonstrates some significance in terms of clinical outcomes, we recommend blinding one of the four WARM steps of the acupressure exercise to further compare each step's efficacy. 

The third limitation is the high attrition rate. The high attrition rate (nearly 50%) of the intervention group is reflective of the real-world experience among people with impaired mobility, particularly in the older population, nonetheless, providing flexible training dates, more phone followup to build up the affinity between the participants and research team, and/or nominal incentives could increase the compliance rate in future studies. There was limited precedent for selecting frequency and duration of acupressure, so when we designed the study, we only had a single dose of acupressure (20 min/day), 5 days a week to estimate its feasibility. It is unknown if a decreased dose would lead to a better compliance. The effectiveness of different protocols will need to be explored in subsequent studies. Future studies should consider assessing the relationship between compliance and the frequency and total length of the acupressure performance at home. It is also important to educate the participants about the potential cumulative impact of acupressure and strongly encourage them to comply with the protocol, as some participants may have high expectations of acupressure in a very short period of time and give up if not observing positive results in the first one or two weeks. On the other hand, a higher retention rate (76%) of the control group indicated the participant's desire of participating in the acupressure training session offered at the end of the study. In the future research, a cross-over design or wait list control group may help to yield more information for a small sample size.

Lastly, the fidelity to treatment of either the participants or the trainer of the intervention was not assessed. The burden of keeping a log at home may have led to the most incomplete logs in our study. Approaches such as weekly or biweekly phone followup that may better assess fidelity as well as increase compliance should be explored in future studies. New technology such as hand-held electronic pain diary may also help to capture more accurate information [36]. As for the trainer fidelity, in addition to training the trainer first as we did in our study, it is recommended to assess the trainers' reliability during mock training sessions if multiple trainers are involved. 

## 5. Conclusions

In conclusion, this preliminary investigation suggests that acupressure exercise can be safely self-administered by women with knee OA and poses no extra harm or risk to the participants. It is highly recommended that future studies use a bigger sample size, appropriate screening criteria, rigorous randomization allocation, and possible blinding to further explore the efficacy of the acupressure protocol on OA symptoms.

## Figures and Tables

**Table 1:** Participants' baseline characteristics.

Participant's characteristics	Acupressure group (*n* = 15)	Control group (*n* = 21)	*P* value
Age	63.47 ± 2.64	59.86 ± 4.91	0.05^a^
Body mass index (BMI)	28.89 ± 4.16	28.46 ± 4.05	0.76^ a^
Pain VAS			
Left knee	4.60 ± 2.64	4.57 ± 2.79	0.98^a^
Right knee	5.00 ± 2.29	5.57 ± 2.29	0.46^a^
Self-rated general health			
Good	66.7%	71.4%	0.94^b^
Satisfactory	26.7%	23.8%	
Not good	6.7%	4.8%	

^a^Independent *t*-test.

^b^Chi-Square test.

**Table 2:** Per-protocol design WOMAC subscale scores and score changes.

WOMAC subscales	Acupressure group (*n* = 8)		Control group (*n* = 16)		Repeated measure	Mann-Whitney
	Score	Score change	Score	Score change	ANOVA, *P* ^a^	*U* test, *P* ^b^
Pain					N/A	
Baseline (0–6 week)	13.75 ± 3.33	− 1.30 ± 2.31	14.44 ± 2.99	− 1.33 ± 2.20		0.91
6 week (6–12 week)	12.63 ± 3.70	0.50 ± 3.42	13.06 ± 3.34	0.44 ± 2.00		0.49
12 week (0–12 week)	13.13 ± 4.49	−0.63 ± 3.93	13.50 ± 3.20	−1.11 ± 2.79		0.78
Stiffness					N/A	
Baseline (0–6 week)	6.75 ± 1.67	−1.60 ± 2.50	6.56 ± 1.03	−0.61 ± 1.69		0.41
6 week (6–12 week)	5.13 ± 2.30	0.50 ± 1.60	5.94 ± 1.77	0.31 ± 1.30		0.45
12 week (0–12 week)	5.63 ± 2.45	−1.13 ± 2.90	6.25 ± 1.65	−0.05 ± 1.39		0.28
Physical function					0.02*	
Baseline (0–6 week)	56.71 ± 16.60	−9.78 ± 11.04	48.93 ± 10.39	−4.18 ± 4.50		0.07
6 week (6–12 week)	43.86 ± 13.06	1.00 ± 9.97	44.40 ± 11.21	1.00 ± 6.55		0.98
12 week (0–12 week)	42.43 ± 15.97	−14.29 ± 8.48	44.93 ± 12.48	−4.61 ± 6.52		0.03*
Total scores					N/A	
Baseline (0–6 week)	77.29 ± 21.48	−13.22 ± 14.17	70.40 ± 13.19	−6.18 ± 7.08		0.24
6 week (6–12 week)	61.00 ± 18.46	2.00 ± 14.60	63.80 ± 15.25	1.75 ± 8.81		0.88
12 week (0–12 week)	59.71 ± 21.42	−17.57 ± 13.40	64.93 ± 16.69	−5.94 ± 8.85		0.07

^a^Repeated measure ANOVA performed only when ANOVA assumptions were met. N/A indicates that ANOVA assumptions were not met.

^b^Mann-Whitney *U* test performed for all subscale score changes.

*P < 0.05.

**Table 3:** Intention-to-treat design WOMAC subscale scores and score changes.

WOMAC subscales	Acupressure group (*n* = 15)		Control group (*n* = 21)		Repeated measure	Mann-Whitney
	Score	Score change	Score	Score change	ANOVA, *P* ^a^	*U* test, *P* ^b^
Pain					0.83	
Baseline (0–6 week)	13.53 ± 2.61	− 0.87 ± 1.96	14.95 ± 3.71	− 1.14 ± 2.08		0.55
6 week (6–12 week)	12.66 ± 2.87	0.27 ± 2.43	13.81 ± 4.18	0.05 ± 2.25		0.49
12 week (0–12 week)	13.93 ± 3.39	−0.60 ± 2.87	13.85 ± 4.05	−1.10 ± 2.64		0.51
Stiffness					0.33	
Baseline (0–6 week)	6.53 ± 1.64	−1.07 ± 2.15	6.77 ± 1.26	−0.37 ± 1.47		0.47
6 week (6–12 week)	5.47 ± 1.96	0.27 ± 1.16	6.38 ± 2.02	0.28 ± 1.15		0.66
12 week (0–12 week)	5.73 ± 2.01	−0.80 ± 2.14	6.67 ± 2.08	−0.10 ± 1.41		0.29
Physical function					0.52	
Baseline (0–6 week)	48.33 ± 15.25	−6.37 ± 9.57	48.85 ± 13.49	−3.54 ± 4.31		0.51
6 week (6–12 week)	42.07 ± 13.33	0.94 ± 7.45	45.47 ± 14.50	−0.46 ± 6.38		0.51
12 week (0–12 week)	43.07 ± 13.13	−5.43 ± 10.17	44.95 ± 13.13	−4.00 ± 6.08		0.98
Total scores					0.95	
Baseline (0–6 week)	68.10 ± 18.34	−8.31 ± 12.47	70.57 ± 17.92	−5.06 ± 6.64		0.80
6 week (6–12 week)	60.20 ± 14.93	1.47 ± 10.88	65.71 ± 20.15	−0.13 ± 8.35		0.53
12 week (0–12 week)	61.73 ± 17.58	−6.83 ± 14.03	65.47 ± 18.52	−5.19 ± 8.34		0.76

^a^Repeated measure ANOVA controlled for age, baseline BMI, and baseline knee pain rating.

^b^Mann-whitney *U* test performed for all subscale score changes.
